# Sedentary behavior and cardiovascular disease risk: An evolutionary perspective

**DOI:** 10.3389/fphys.2022.962791

**Published:** 2022-07-27

**Authors:** Simon Higgins, Alexander Pomeroy, Lauren C. Bates, Craig Paterson, Bethany Barone Gibbs, Herman Pontzer, Lee Stoner

**Affiliations:** ^1^ Department of Exercise and Sport Science, University of North Carolina at Chapel Hill, Chapel Hill, NC, United States; ^2^ Department of Epidemiology and Biostatistics, West Virginia University, Morgantown, WV, United States; ^3^ Duke Global Health Institute, Duke University, Durham, NC, United States; ^4^ Department of Epidemiology, Gillings School of Global Public Health, University of North Carolina at Chapel Hill, Chapel Hill, NC, United States

**Keywords:** sitting, cardiovascular disease, hunter-gatherer, Hazda, public health policy

## Abstract

A ubiquitous aspect of contemporary societies is sedentary behavior (SB), defined as low intensity activities in a seated, reclined, or supine posture. Leading public health agencies, including the World Health Organization, have recognized the strong association between SB and poor health outcomes, particularly cardiovascular disease. However, while public health agencies have begun to advocate for “reductions” in SB, the current US guidelines are typically vague and non-specific. There is good reasoning behind this non-committal advocacy—there is limited mechanistic and clinical evidence to support policy development. To guide SB policy development, it is important to first consider the origins and evolution of SB, including the following: 1) is SB really a novel/contemporary behavior? i.e., how has this behavior evolved? 2) how did our ancestors sit and in what contexts? 3) how does SB interact with 24-hour activity behaviors, including physical activity and sleep? 4) what other historical and contemporary facets of life interact with SB? and 5) in what context do these behaviors occur and how might they provide different evolutionarily novel stressors? This perspective article will synthesize the available evidence that addresses these questions and stimulate discussion pertaining to the lessons that we can learn from an historical and evolutionary perspective. Last, it will outline the gaps in current SB interruption literature that are hindering development of feasible SB reduction policy.

## 1 Introduction: Defining sedentary behavior

Over the past decade, the field of sedentary behavior (SB) research has expanded and evidence supporting a direct association between SB and cardiovascular disease (CVD) has emerged. However, relative to literature examining the cardioprotective effects of physical activity, SB-based literature is nascent. (Gibbs et al., 2015) Additionally, widespread confusion has resulted from the terms “sedentary” and “physical inactivity” being used interchangeably despite the fact that these terms relate to biologically novel constructs. (Gibbs et al., 2015) Therefore, it is critical to begin this discussion by defining and differentiating these terms (Table 1). SB is a low intensity behavior (<1.5 metabolic equivalents, METs) in a seated, reclined, or supine posture (Gibbs et al., 2015) whereas, physical inactivity describes the habitual daily activity for someone who does not meet the recommended guidelines (i.e., 150 min per week for US adults) for moderate-to-vigorous intensity physical activity (MVPA). (van der Ploeg and Hillsdon, 2017; Stoner et al., 2021) In industrialized societies, a majority of SB is accrued in a seated posture, while at home, work, or during motorized transportation. This includes behaviors such as watching television or other screen-based entertainment, eating meals, completing tasks on computers and smartphones, and driving to and from work. In this paper, we take an evolutionary perspective on this issue. We will begin by discussing how and why SB is biologically novel to physical [in]activity, discuss the proposed mechanisms for the negative cardiovascular effects of prolonged SB, hypothesize about the SB performed by our ancestors thousands of years ago and compare that with modern day, when technology and convenience have taken prominence over survival and necessity, and bring this together with discussion of current public policy and future directions.

**TABLE 1:** Key concepts—definitions and caveats.

Concept	Definition	Caveats
24-hour activity cycle (24-HAC)	Activities conducted over a 24-hour period including, sleep, sedentary behavior, light-intensity physical activity, and moderate-to-vigorous intensity physical activity.^1^	These behaviors are interrelated and must be considered together when examining the impact of behavioral interventions or public policy.
Exercise	A subset of physical activity, that is, planned, structured, and repetitive.^1^	N/A
Light intensity physical activity (LPA)	Physical activities requiring energy expenditures of 1.5 to < 3 METs.	N/A
Metabolic equivalent (MET)	Unit used to describe the absolute intensity of physical activity. A ratio of your working metabolic rate relative to your resting metabolic rate.^1^	N/A
Moderate-to-vigorous intensity physical activity (MVPA)	Physical activities requiring energy expenditures of ≥ 3 METs.	N/A
Physical activity	Any bodily movement produced by skeletal muscles that requires energy expenditure.^1^	This includes both exercise and non-exercise activity thermogenesis. Physical activity is typically described across domains such as transportation, occupational, leisure-time, etc.
Physical inactivity	An insufficient level of moderate-to-vigorous intensity physical activity level to meet present physical activity recommendations.^1^	An individual who is physically inactive is often wrongly labelled as “sedentary”.
Sedentary behavior (SB)	Any waking behavior characterized by an energy expenditure < 1.5 METs, while in a seated, reclined, or supine posture.^1^	The typical definition does not include quiet standing or standing-related behaviors with low energy expenditure, nor does it include squatting or kneeling behaviors such as those commonly seen in hunter-gatherer communities.
Sleep	Sleep is a recurring, reversible neuro-behavioral state of relative perceptual disengagement from and unresponsiveness to the environment. Sleep is typically accompanied (in humans) by postural recumbence, behavioral quiescence, and closed eyes.^2^	Sleep time does not include time in bed while awake. Sleep also includes many different characteristics, with simple assessments of duration being an oversimplification of its impact on health, as well as the impact of other activity behaviors on sleep.
Standing	A stationary, upright posture, supported on one or two legs.	Considered separate from SB, though energy expenditure likely does not classify as LPA.

Several definitions were previously published in ^1^Stoner L, Barone Gibbs B, Meyer ML, Fryer S, Credeur D, Paterson C, et al. A Primer on Repeated Sitting Exposure and the Cardiovascular System: Considerations for Study Design, Analysis, Interpretation, and Translation. Frontiers in Cardiovascular Medicine. 2021; 8 and ^2^Carskadon MA, Dement WC. Normal human sleep: an overview. In: Kryger MH, Roth T, Dement WC, editors. Principles and practice of sleep medicine. 4th ed. Philadelphia, PA: elsevier saunders; 2005. pp. 13–23.

24-HAC, 24-hour activity cycle; SB, sedentary behavior; PA, physical activity; LPA, light intensity physical activity; MVPA, moderate-to-vigorous intensity physical activity; MET, metabolic equivalent.

## 2 Proposed mechanisms of sedentary behavior-induced cardiovascular pathophysiology

The primary driver for the deleterious cardiovascular effects of SB is thought to be venous pooling within the lower limbs as a consequence of reduced muscle activity and therefore reduced action of the muscle pump. (Stoner et al., 2021) The subsequent venous pooling is hypothesised to reduce venous return and, in turn, stroke volume. (Horiuchi and Stoner, 2021) This reduction in stroke volume, in tandem with increased hydrostatic pressure within the lower limbs (Padilla et al., 2009) and increased arterial tortuosity, creates a unique haemodynamic environment whereby cardiovascular burden may be increased. (Walsh et al., 2017) Our group has demonstrated that acute bouts of sitting can lead to impaired lower limb vascular function, (Paterson et al., 2020) increased peripheral blood pressure, (Paterson et al., 2021) and increased central and peripheral arterial stiffness. (Evans et al., 2019) Additionally, repeated exposure to acute bouts of sitting may also impair glucose and triglyceride metabolism via a downregulation of skeletal muscle contraction-mediated glucose uptake and lipoprotein lipase, respectively. (Loh et al., 2020) The downregulation of these metabolic pathways is thought to increase systemic inflammation, which may further impair vascular function and contribute to increased cardiovascular burden. Whilst the long-term effects of these acute detriments to cardiovascular function are unknown, it is conceivable that repeated exposure to these physiological insults may, over the course of the lifetime, contribute to the observed relationship between high SB and CVD risk.

Regularly interrupting bouts of prolonged SB with physical activity appears to offset the acute detriments of prolonged SB via the maintenance of the muscle pump and blood flow. (Paterson et al., 2020; Paterson et al., 2021) However, the specific type, frequency, and duration of interruptions to SB that can be easily integrated into modern lifestyles and are effective in preventing the aforementioned pathophysiological cascade are largely unknown. One guide for these interventions is the activity behaviour of farming and foraging communities, who experience excellent heart health and low CVD incidence into old age. (Kaplan et al., 2017; Pontzer et al., 2018; Gurven and Lieberman, 2020) These populations are much more physically active than industrialized populations (Pontzer et al., 2018; Gurven and Lieberman, 2020) and provide a model for the activity behaviours common among our evolutionary past. Insights from these communities may help to explain why SB, as we know it today, is a relatively “novel” societal challenge in industrialized populations, whilst highlighting opportunities for the development of interruption strategies.

## 3 Is sedentary behavior really a novel problem?

Humans are primates, part of the hominoid, or ape, family. Roughly 7 million years ago, our lineage, the hominins, split from that of chimpanzees and bonobos, our closest living relatives. The earliest hominins walked on two legs but, based on their reconstructed diet and body size, were probably like living apes in their daily physical activity, traveling only modest distances to forage and resting up to 10 h per day. (Pontzer, 2017) About 2.5 million years ago, the archeological record documents a major shift in hominin foraging strategies. Cut marked animal bones and a proliferation of stone tools indicates the addition of hunted and scavenged game as a substantial part of the hominin diet. (Domínguez-Rodrigo et al., 2005; Plummer and Finestone, 2017) This behavioral shift coincides with changes in anatomy, including increased brain size and smaller teeth, that mark the origins of the genus *Homo*. Hunting and gathering continued to shape the human genus for over 2 million years.

Our species, *Homo sapiens*, emerged in Africa about 300,000 years ago, (Hublin et al., 2017) and all human populations hunted and gathered until the origins of farming just 12,000 years ago. (Pontzer and Wood, 2021) Hunting and gathering is physically demanding. The high-quality foods humans target, including wild game, require a great deal of work to acquire, far more than other apes perform. (Kraft et al., 2021) Subsistence farming, which replaced hunting and gathering in most of the world as the dominant subsistence strategy, is equally strenuous. Among subsistence farming and hunter-gatherer societies living today, men and women regularly get 1–2 h of MVPA and over 12,000 steps per day. (Pontzer et al., 2018; Kraft et al., 2021; Wood et al., 2021) Only very recently, with widespread industrialization and mechanization beginning in the late 1700’s and early 1800’s, have the physical demands of daily life been substantially reduced. (Gurven and Lieberman, 2020; Pontzer, 2021)

In the physically demanding lifestyles typical of our hunter-gatherer and farming past, energy minimizing strategies, such as what we define today as SB, would have been important for conserving energy. In fact, among hunter-gatherer communities today, time spent in SB does not significantly differ from estimates of modernized societies. (Raichlen et al., 2020; O’Brien et al., 2022) However, the type of SB typical in these populations differs substantially from that of industrialized societies. (Raichlen et al., 2020) With the mass production of chairs, most SB in industrialized populations involves sitting, whether at work, while socializing, or in most forms of mechanized transportation. (Owen et al., 2011; Henson et al., 2016) Whilst sedentary time in industrialized societies is largely occupied by bouts of chair sitting, it appears that sedentary time for our ancestors may have been largely occupied by alternatives such as squatting, kneeling, and sitting on the ground. This is an important consideration as these postures, whilst broadly fitting the definition of SB in terms of energy expenditure or posture, involve greater lower limb muscle activity. By maintaining lower limb muscle activity, it may be possible to reduce venous pooling and thus offset the deleterious effects of SB on the vascular system. Thus, while our ancestors likely engaged in high amounts of SB, the type of SB was likely different (i.e., squatting not sitting) reducing the impeding disease risk associated with the SB of today. Furthermore, in industrialized societies SB is multidimensional, meaning that it is a behavior that occurs in different contexts, with a variety of other behaviors occurring simultaneously. (Owen et al., 2011) The co-occurring behaviors associated with modern-day SB, such as stress-inducing computer work or snacking, (Lee and Kim, 2019; Mattioli et al., 2020) are also different than our ancestors (e.g., preparing food, crafting tools, resting in community). Longevity has also increased with improved sanitation and medicine in developed economies, with more individuals living into their 70 and 80s, and this longer lifespan allows for more cumulative exposure to SB and these co-occurring behaviors, affording greater opportunity for the manifestation of chronic disease.

## 4 Characteristics of contemporary sedentary behavior

### 4.1 The evolution of sedentary behavior context

In past and contemporary farming and foraging populations, bouts of SB are often coupled with activities such as crafting tools or resource refinement, (Jones, 1996) consisting of a singular, high attentional demand, and likely performed in relatively quiet environments. Furthermore, SB in these contexts may have been more frequently interrupted with bouts of MVPA, reducing the negative impact of SB. In the present day, people deal with varied and complex stressors during bouts of SB, such as completing high-focus tasks in a noisy workplace or driving motorized vehicles on busy roads. Similarly, leisure time SB has increased and is often characterized by long duration uninterrupted bouts, (Aadahl et al., 2013) which may be related to increased options for sedentary leisure time activities such as television watching and other screen-based entertainment. This combination of stressful workplace SB and increased volumes of leisure time SB contrasts with the experience of historic humans. Thus, it could be said that the SB-related stressors experienced by our ancestors seem to be acute in nature, likely related to activity interruptions, whereas the stressors associated with SB in modern society are chronic in nature. Exploring the different contexts that SB is performed in along with the context-specific co-occurring behaviors may give insight into potential barriers to SB change and policy generation.

### 4.2 Co-occurring behaviors

#### 4.2.1 Sedentary behavior in the workplace

The environment for the typical working adult has become increasingly convenience-based, particularly within the past century, (Aadahl et al., 2013; Clark and Sugiyama, 2015) which has led to increased daily durations of SB and associated risk of CVD and mortality. (Ekelund et al., 2019) Additionally, people are performing high stress tasks while seated, such as typing reports or writing emails in disruptive or busy environments or with demanding deadlines; tasks that require high focus. Research on mental stress reactivity, or the degree to which stressful tasks disrupt the cardiovascular system, is one link between SB and CVD risk. Individuals with higher mental stress reactivity have a greater number of CVD risk factors, such as incident hypertension and increased carotid intima-media thickness. (Chida and Steptoe, 2010; Carroll et al., 2012) These acute stressors may cause impairments to cardiovascular function that compound over time and eventually manifest as increased CVD incidence. Furthermore, many companies are now supporting hybrid or remote working environments following the COVID-19 pandemic. While these working environments are associated with decreased time spent travelling to and from the office, the increased number of days working from home have been associated with increased work-related and total SB, and a decreased MVPA. (Javad Koohsari et al., 2021) This combination of prolonged work-related SB time and acute mental stress may account for many of the negative cardiovascular impacts of SB in this context. While interrupting workplace SB may be logistically challenging due to work-related demands, feasible interruption strategies could have a high yield for reducing disease burden and may be easily adoptable in hybrid/remote environments.

#### 4.2.2 Sedentary behavior during transportation

The nature of transport for both work and leisure has changed in modern times. For much of human history, walking was the primary mode of transportation. However, since the adoption of the automobile, rates of walking to work have decreased. Between 1980 and 2012, rates of walking to work fell by half. (McKenzie, 2014) This trend may have public health impact, as individuals walking to work are twice as likely to be meeting physical activity guidelines. (Barnett et al., 2019) Moreover, the adoption of automobiles as transport has exposed people to higher doses of SB and time spent sitting in automobiles has been identified as a risk for premature mortality. (Owen et al., 2010) A study investigating the effect of a 2-hour simulated commute through metropolitan traffic reported 21.3% reductions in the standard deviation of the N-N intervals (SDNN), a measure of heart rate variability. (Sarnat et al., 2014) Such depressions in cardiovascular autonomic function, likely a combination of reduced vagal tone (primarily) and increased sympathetic stimulation (secondarily), (Shaffer and Ginsberg, 2017) are associated with increased risk for CVD. As automobiles provide a challenging environment for which to provide SB guidelines, intervention strategy research may be more suited to determining when driving can be replaced with more physically active transport strategies, such as walking or bicycling.

#### 4.2.3 Sedentary behavior during leisure time

Norms around SB during mealtimes and recreational leisure time have also changed. While little is known about the impact or feasibility of interrupting mealtime SB, literature suggests that meals in the modern day have become increasingly nutrient dense. The typical Western diet is relatively high in both fat and refined carbohydrate content compared to diets in previous eras, (Drewnowski and Popkin, 1997) and the consumption of a high-fat meal before a sitting bout is associated with increased markers of CVD risk for up to 180 min after the meal. (Fryer et al., 2021) While similar research has yet to be conducted on SB and high-carbohydrate meals, some data indicates that high-carbohydrate meals followed by prolonged sitting result in elevated postprandial blood glucose responses relative to interrupted sitting, which may have cardiovascular implications. (Dempsey et al., 2018) Similarly, while the behaviors of historic humans after mealtimes may be uncertain, technology has increased the options of leisure time SB following mealtimes available to modern humans. Television viewing time is one of the most common forms of leisure time SB, and is commonly used as a proxy for SB in scientific analyses. (Owen et al., 2010) For example, one study found that ≥4 h of average daily television time was associated with increased CVD mortality, with each additional hour conveying extra risk. (Dunstan et al., 2010) While in recent history people may have engaged in forms of SB after meal times, such as playing games with others or resting and conversing, the television does not require the same level of social engagement. This limited engagement may contribute to lower total daily energy expenditure and encourage other potentially detrimental behaviors such as snacking on energy-dense foods. (Thomson et al., 2008) Accordingly, understanding the nature of modern SB in the context of other lifestyle behaviors and activity domains may provide opportunity to identify the most effective and feasible SB interruption strategies.

## 5 Contemporary sedentary behaviors as part of the 24-hour activity cycle

### 5.1 Interrelationships among 24-hour activity cycle behaviors

Physical inactivity is a challenge faced ubiquitously across developed nations, with a 2012 analysis suggesting that it is directly responsible for 5.1–12.5% of mortality worldwide. (Lee et al., 2012) Accordingly, the promotion of physical activity has been a major focus of public health campaigns for over 70 years. Recent campaigns have focused on increasing energy expenditure through any kind of activity [e.g., the Move Your Way® campaign from the US Department of Health and Human Services (U.S. Department of Health and Human Services, 2022)] to reduce the risk of obesity-related chronic disease. However, it is important to remember that time spent “physically active”, especially in MVPA, comprises a relatively small percentage of each 24-hour day, whereas over 50% of the waking day is spent sedentary. Moreover, one can be physically active, meeting current public health guidelines for time spent in MVPA, but still be highly sedentary. College students are great examples of this, often performing 7–10 h of SB daily, (Castro et al., 2020) despite meeting physical activity guidelines more frequently than other age groups within the US population. (Ward et al., 2015) Importantly, the potential for interrupting prolonged SB with bouts of standing and light-intensity physical activity, which can be completed in a much greater volume than MVPA, cannot be missed. Indeed, evidence supports that the health benefits of accumulating these behaviors across the day can become quite meaningful with higher levels of engagement. (Chastin et al., 2019; Gibbs et al., 2021)

Though 24-hour activity cycle (24-HAC) behaviors such as physical activity and SB are behaviorally and physiologically independent, it is important to remember that they are interrelated. Efforts to change one behavior likely impact other aspects of the 24-HAC, either positively or in a compensatory manner. Examples of the interrelated nature of 24-HAC behaviors include, inverse associations between SB and physical activity in adults, with associations of small to moderate magnitude reported between SB and MVPA and moderate to large magnitude between SB and light intensity physical activity. (Mansoubi et al., 2014) Similarly, SB is associated with sleep characteristics including sleep duration. In a sample of over 6,000 European adults, individuals who were classified as both short and long sleepers (<6 h or >9 h, respectively) spent a significantly higher proportion of their waking time in SB. The short sleepers also tended to spend ∼26.5 min/day more in front of a screen. (Lakerveld et al., 2016) Such relationships among 24-HAC behaviors are also bidirectional, with changes in activity behaviors both affecting and being affected by changes in the other aspects of the 24-HAC. (Imes et al., 2021) This complicated landscape highlights the need to consider activity behaviors together when designing research and developing public health guidelines.

## 6 Discussion

### 6.1 Key messages and public policy

**FIGURE 1:**
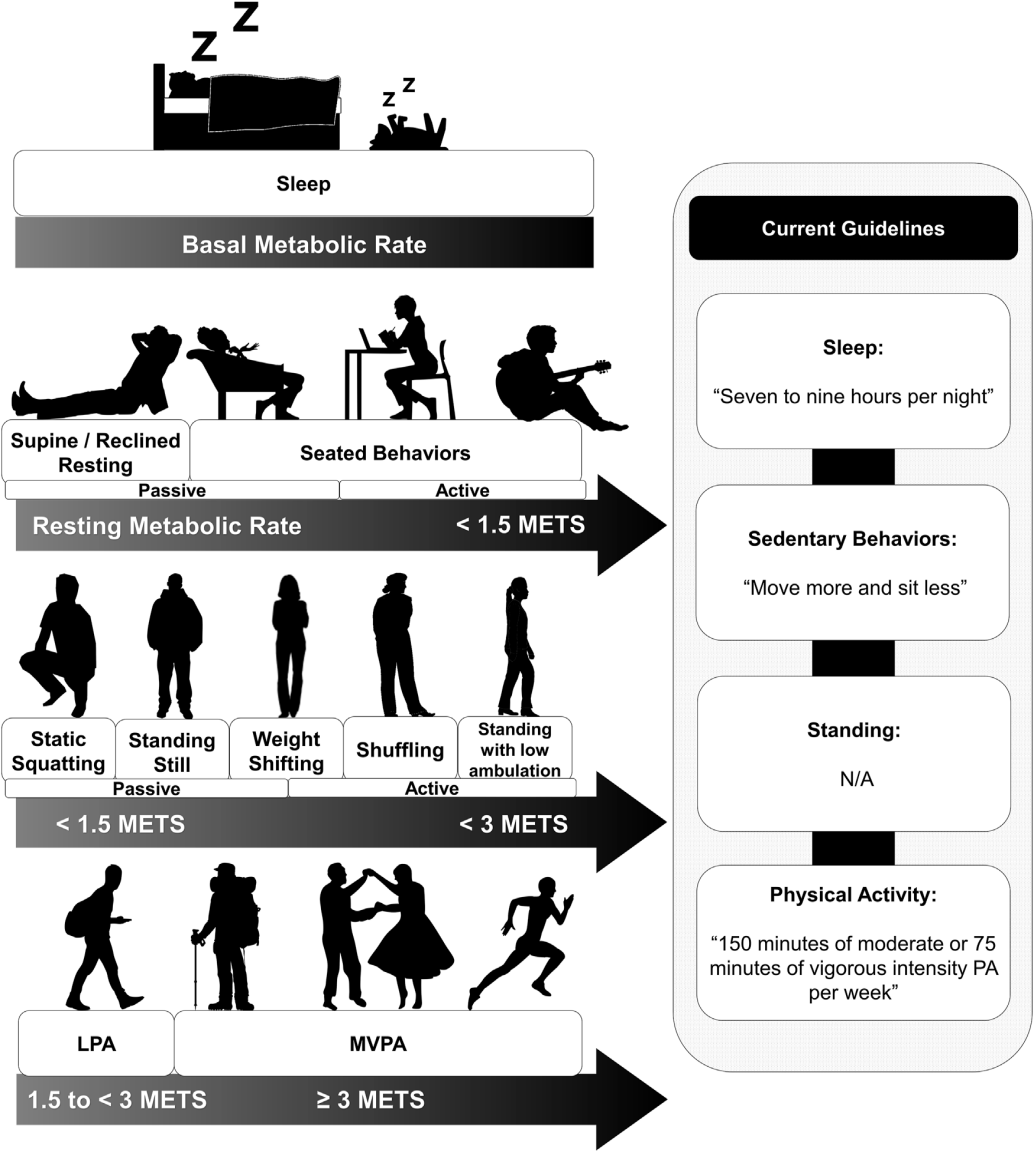
Energy expenditure continuums and current guidelines for activity behaviors, including sleep, sedentary behaviors, standing behaviors, and physical activities.

In this section we focus on the US, which is the home of the authors. However, the arguments are mostly generalizable. Current US public health activity guidelines focus on achieving 150 min of moderate intensity exercise weekly (Figure 1), with no integrated guidance on sleep and little emphasis on the importance of SB reduction. Clear guidelines regarding the total “safe” duration of SB or potentially harmful context-specific behaviors (e.g., television viewing) are also absent. (U.S. Department of Health and Human Services; Office of Disease Prevention and Health Promotion, 2018) To receive guidance on all 24-HAC behaviors, the American public must look to multiple sources (e.g., National Science Foundation guidelines for sleep duration (Hirshkowitz et al., 2015), which may contribute to confusion and present a barrier to adherence. Comprehensive activity guideline models do exist, with the Canadian 24-hour movement guidelines being a good example. (Canadian Society for Exercise Physiology, 2021) However, excluding the aforementioned Canadian guidelines, current SB guidelines are typically limited to “sit less”. This includes recent global guidelines from the WHO, who suggest that all populations should “limit the amount of time spent being sedentary” and “replace SB with physical activity of any intensity”. (Bull et al., 2020) Such public health messaging is likely to have minimal effect on behavior change. To have any hope of moving the needle, i.e., to change behavior at a public health level, we need reputable sources to disseminate clear yet simple messaging using platforms capable of reaching the masses. However, before comprehensive 24-HAC guidelines can be published in the US, there are several research gaps surrounding SB that must first be filled—some which are discussed in the following section. Ultimately, behavior change is challenging and typically requires multi-level strategies, including strategies which facilitate behavior change at physical environment, inter-individual, and intra-individual levels.

### 6.2 Future directions

In the this section we detail the considerations made by the US Preventive Services Task Force (USPSF) when developing policy for primary care clinicians. (Harris et al., 2001) Similar to the processes used by many evidence-based groups, the USPTF systematically reviews the research literature and grades the strength of the evidence, assigning a grade ranging: from A (strongly recommended) to D (recommend against), or I (insufficient evidence). The evidence includes: 1) individual studies (study design, internal validity); 2) the linkage between each key question (aggregate internal validity, aggregate external validity, coherence/consistency); and 3) whether the evidence is adequate to determine the existence and magnitude of a causal connection between the preventive service and health outcomes. With respect to 1) grading individual studies, randomized controlled trial (RCT) cohort studies are typically considered the strongest, though internal and external validity are graded separately. With respect to 2) the linkage between each key question, the USPSTF considers aggregate internal and external validity, and the coherence of the body of evidence. Last, the USPSTF considers 3) whether the evidence is adequate to determine the existence and magnitude of a causal connection between the preventive service and health outcomes. This includes assessing the magnitude of net benefit, in other words, do the benefits outweigh potential harms? Additionally, in accordance with other agencies, the USPSTF considers the likelihood of a biologically plausible mechanism between cause and effect. (Harris et al., 2001; Becker et al., 2017; Dailey et al., 2018).

**FIGURE 2:**
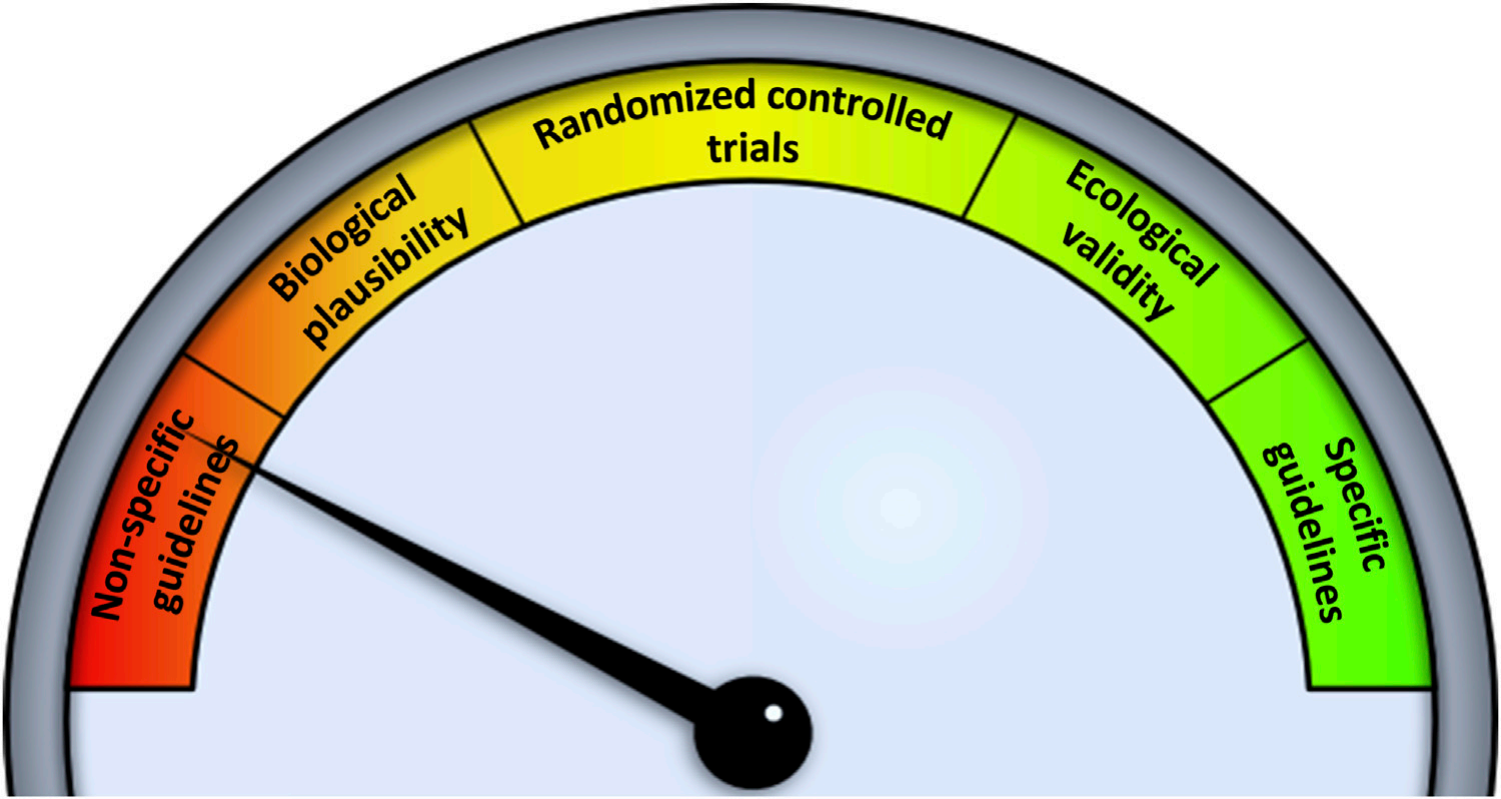
“Moving the needle” on current sedentary behavior research towards policy generation.

The USPSTF has previously developed policy for “Healthful Diet and Physical Activity for Cardiovascular Disease Prevention in Adults With Cardiovascular Risk Factors: Behavioral Counseling”. (US Preventive Services Task Force, 2014) This physical activity policy received a B grade—a recommendation that the service is routinely provided. The B grade was supported by a deep body of research. The first physical activity study was conducted in the 1950s, (Morris et al., 1953) and since this time many well-controlled (internally-valid) RCTs have provided ample evidence to support net benefit to cardiovascular health. (Katzmarzyk et al., 2019) The SB-based literature is currently void of well-designed RCTs that test mechanism-informed SB interruption strategies. In turn, to facilitate the design of RCTs we need to better understand the effects of repeated exposure to uninterrupted prolonged SB on the cardiovascular system. To move the policy needle (Figure 2), we need studies with high internal validity to assess mechanisms (biological plausibility) and to identify SB interruption strategies that directly tackle the mechanisms of action. Establishing biological plausibility will facilitate the design of well-controlled RCTs that can ultimately support high-level summaries such as meta-analyses. Last, to support USPSTF’s third criteria, i.e., a causal connection between the preventive service and health outcomes, we need to consider that laboratory-based studies, or even well-controlled RCTs, may lack ecological validity. Ecological validity tells us whether the findings can be generalized to real-world or naturalistic settings. Currently, we know little about how SB interacts with 24-HAC and other behaviors, or the importance of the SB context (e.g., occupation, leisure computer, television, transportation).

At present, we are some ways from developing specific SB guidelines. However, if we as a research community consider the steps necessary to design well-controlled SB reduction studies, while contemplating the criteria used to aid policy development, we can accelerate guideline development.

## Data Availability

The original contributions presented in the study are included in the article/supplementary material, further inquiries can be directed to the corresponding author.

## References

[B1] Aadahl M. Andreasen A. H. Hammer-Helmich L. Buhelt L. Jørgensen T. Glümer C. (2013). Recent temporal trends in sleep duration, domain-specific sedentary behaviour and physical activity. A survey among 25-79-year-old Danish adults. Scand. J. Public Health 41 (7), 706–711. 10.1177/1403494813493151 23798478

[B2] Barnett A. Akram M. Sit C. H. Mellecker R. Carver A. Cerin E. (2019). Predictors of healthier and more sustainable school travel mode profiles among Hong Kong adolescents. Int. J. Behav. Nutr. Phys. Act. 16 (1), 48. 10.1186/s12966-019-0807-4 31138203 PMC6537196

[B3] Becker R. A. Dellarco V. Seed J. Kronenberg J. M. Meek B. Foreman J. (2017). Quantitative weight of evidence to assess confidence in potential modes of action. Regul. Toxicol. Pharmacol. 86, 205–220. 10.1016/j.yrtph.2017.02.017 28232103

[B4] Bull F. C. Al-Ansari S. S. Biddle S. Borodulin K. Buman M. P. Cardon G. (2020). World Health Organization 2020 guidelines on physical activity and sedentary behaviour. Br. J. Sports Med. 54 (24), 1451–1462. 10.1136/bjsports-2020-102955 33239350 PMC7719906

[B5] Canadian Society for Exercise Physiology (2021). Canadian 24-hour movement guidelines: An integration of physical activity. *Sedentary Behaviour, and Sleep* [Online]. Canadian Society for Exercise Physiology. Available at: https://csepguidelines.ca/ .

[B6] Carroll D. Ginty A. T. Der G. Hunt K. Benzeval M. Phillips A. C. (2012). Increased blood pressure reactions to acute mental stress are associated with 16-year cardiovascular disease mortality. Psychophysiology 49 (10), 1444–1448. 10.1111/j.1469-8986.2012.01463.x 22958235

[B7] Castro O. Bennie J. Vergeer I. Bosselut G. Biddle S. J. H. (2020). How sedentary are university students? A systematic review and meta-analysis. Prev. Sci. 21 (3), 332–343. 10.1007/s11121-020-01093-8 31975312

[B8] Chastin S. F. M. De Craemer M. De Cocker K. Powell L. Van Cauwenberg J. Dall P. (2019). How does light-intensity physical activity associate with adult cardiometabolic health and mortality? Systematic review with meta-analysis of experimental and observational studies. Br. J. Sports Med. 53 (6), 370–376. 10.1136/bjsports-2017-097563 29695511 PMC6579499

[B9] Chida Y. Steptoe A. (2010). Greater cardiovascular responses to laboratory mental stress are associated with poor subsequent cardiovascular risk status: A meta-analysis of prospective evidence. Hypertension 55 (4), 1026–1032. 10.1161/hypertensionaha.109.146621 20194301

[B10] Clark B. Sugiyama T. (2015). “Prevalence, trends, and correlates of sedentary behavior,” in Physical activity, exercise, sedentary behavior and health. Editors Kanosue K. Oshima S. Cao Z.-B. Oka K. (Tokyo: Springer Japan), 79–90.

[B11] Dailey J. Rosman L. Silbergeld E. K. (2018). Evaluating biological plausibility in supporting evidence for action through systematic reviews in public health. Public Health 165, 48–57. 10.1016/j.puhe.2018.08.015 30368168 PMC6289655

[B12] Dempsey P. C. Larsen R. N. Winkler E. A. H. Owen N. Kingwell B. A. Dunstan D. W. (2018). Prolonged uninterrupted sitting elevates postprandial hyperglycaemia proportional to degree of insulin resistance. Diabetes Obes. Metab. 20 (6), 1526–1530. 10.1111/dom.13254 29431272

[B13] Domínguez-Rodrigo M. Pickering T. R. Semaw S. Rogers M. J. (2005). Cutmarked bones from pliocene archaeological sites at gona, Afar, Ethiopia: Implications for the function of the world's oldest stone tools. J. Hum. Evol. 48 (2), 109–121. 10.1016/j.jhevol.2004.09.004 15701526

[B14] Drewnowski A. Popkin B. M. (1997). The nutrition transition: New trends in the global diet. Nutr. Rev. 55 (2), 31–43. 10.1111/j.1753-4887.1997.tb01593.x 9155216

[B15] Dunstan D. W. Barr E. L. Healy G. N. Salmon J. Shaw J. E. Balkau B. (2010). Television viewing time and mortality: The Australian diabetes, obesity and lifestyle study (AusDiab). Circulation 121 (3), 384–391. 10.1161/circulationaha.109.894824 20065160

[B16] Ekelund U. Brown W. J. Steene-Johannessen J. Fagerland M. W. Owen N. Powell K. E. (2019). Do the associations of sedentary behaviour with cardiovascular disease mortality and cancer mortality differ by physical activity level? A systematic review and harmonised meta-analysis of data from 850 060 participants. Br. J. Sports Med. 53 (14), 886–894. 10.1136/bjsports-2017-098963 29991570

[B17] Evans W. S. Stoner L. Willey Q. Kelsch E. Credeur D. P. Hanson E. D. (2019). Local exercise does not prevent the aortic stiffening response to acute prolonged sitting: A randomized crossover trial. J. Appl. Physiol. 127 (3), 781–787. 10.1152/japplphysiol.00318.2019 31318613

[B18] Fryer S. Stone K. Paterson C. Brown M. Faulkner J. Lambrick D. (2021). Central and peripheral arterial stiffness responses to uninterrupted prolonged sitting combined with a high-fat meal: A randomized controlled crossover trial. Hypertens. Res. 44 (10), 1332–1340. 10.1038/s41440-021-00708-z 34334790 PMC8490151

[B19] Gibbs B. B. Diaz K. M. Kowalsky R. J. Smith P. M. Stoner L. (2021). Association of standing with cardiovascular disease and mortality in adults. Curr. Epidemiol. Rep. 8 (4), 200–211. 10.1007/s40471-021-00276-3

[B20] Gibbs B. B. Hergenroeder A. L. Katzmarzyk P. T. Lee I. M. Jakicic J. M. (2015). Definition, measurement, and health risks associated with sedentary behavior. Med. Sci. Sports Exerc. 47 (6), 1295–1300. 10.1249/mss.0000000000000517 25222816 PMC4362881

[B21] Gurven M. D. Lieberman D. E. (2020). WEIRD bodies: Mismatch, medicine and missing diversity. Evol. Hum. Behav. 41 (5), 330–340. 10.1016/j.evolhumbehav.2020.04.001 33100820 PMC7584376

[B22] Harris R. P. Helfand M. Woolf S. H. Lohr K. N. Mulrow C. D. Teutsch S. M. (2001). Current methods of the US preventive services task Force: A review of the process. Am. J. Prev. Med. 20 (3), 21–35. 10.1016/s0749-3797(01)00261-6 11306229

[B23] Henson J. Dunstan D. W. Davies M. J. Yates T. (2016). Sedentary behaviour as a new behavioural target in the prevention and treatment of type 2 diabetes. Diabetes. Metab. Res. Rev. 32 (1), 213–220. 10.1002/dmrr.2759 26813615

[B24] Hirshkowitz M. Whiton K. Albert S. M. Alessi C. Bruni O. DonCarlos L. (2015). National sleep foundation's sleep time duration recommendations: Methodology and results summary. Sleep. Health 1 (1), 40–43. 10.1016/j.sleh.2014.12.010 29073412

[B25] Horiuchi M. Stoner L. (2021). Effects of compression stockings on lower-limb venous and arterial system responses to prolonged sitting: A randomized cross-over trial. Vasc. Med. 26 (4), 386–393. 10.1177/1358863x20988899 33606965

[B26] Hublin J. J. Ben-Ncer A. Bailey S. E. Freidline S. E. Neubauer S. Skinner M. M. (2017). New fossils from Jebel Irhoud, Morocco and the pan-African origin of *Homo sapiens* . Nature 546 (7657), 289–292. 10.1038/nature22336 28593953

[B27] Imes C. C. Bizhanova Z. Kline C. E. Rockette-Wagner B. Chasens E. R. Sereika S. M. (2021). Bidirectional relationship between sleep and sedentary behavior in adults with overweight or obesity: A secondary analysis. Sleep. Adv. 2 (1), zpab004. 10.1093/sleepadvances/zpab004 33870194 PMC8038645

[B28] Javad Koohsari M. Nakaya T. Shibata A. Ishii K. Oka K. (2021). Working from home after the COVID-19 pandemic: Do company employees sit more and move less? Sustainability 13 (2), 939. 10.3390/su13020939

[B29] Jones T. L. (1996). Mortars, pestles, and division of labor in prehistoric California: A view from big sur. Am. Antiq. 61 (2), 243–264. 10.2307/282420

[B30] Kaplan H. Thompson R. C. Trumble B. C. Wann L. S. Allam A. H. Beheim B. (2017). Coronary atherosclerosis in indigenous south American tsimane: A cross-sectional cohort study. Lancet 389 (10080), 1730–1739. 10.1016/s0140-6736(17)30752-3 28320601 PMC6028773

[B31] Katzmarzyk P. T. Powell K. E. Jakicic J. M. Troiano R. P. Piercy K. Tennant B. (2019). Sedentary behavior and health: Update from the 2018 physical activity guidelines advisory committee. Med. Sci. Sports Exerc. 51 (6), 1227–1241. 10.1249/mss.0000000000001935 31095080 PMC6527341

[B32] Kraft T. S. Venkataraman V. V. Wallace I. J. Crittenden A. N. Holowka N. B. Stieglitz J. (2021). The energetics of uniquely human subsistence strategies. Science 374 (6575), eabf0130. 10.1126/science.abf0130 34941390

[B33] Lakerveld J. Mackenbach J. D. Horvath E. Rutters F. Compernolle S. Bárdos H. (2016). The relation between sleep duration and sedentary behaviours in European adults. Obes. Rev. 17 (S1), 62–67. 10.1111/obr.12381 26879114

[B34] Lee E. Kim Y. (2019). Effect of University students' sedentary behavior on stress, anxiety, and depression. Perspect. Psychiatr. Care 55 (2), 164–169. 10.1111/ppc.12296 29797324 PMC7818186

[B35] Lee I. M. Shiroma E. J. Lobelo F. Puska P. Blair S. N. Katzmarzyk P. T. (2012). Effect of physical inactivity on major non-communicable diseases worldwide: An analysis of burden of disease and life expectancy. Lancet 380 (9838), 219–229. 10.1016/s0140-6736(12)61031-9 22818936 PMC3645500

[B36] Loh R. Stamatakis E. Folkerts D. Allgrove J. E. Moir H. J. (2020). Effects of interrupting prolonged sitting with physical activity breaks on blood glucose, insulin and triacylglycerol measures: A systematic review and meta-analysis. Sports Med. 50 (2), 295–330. 10.1007/s40279-019-01183-w 31552570 PMC6985064

[B37] Mansoubi M. Pearson N. Biddle S. J. Clemes S. (2014). The relationship between sedentary behaviour and physical activity in adults: A systematic review. Prev. Med. 69, 28–35. 10.1016/j.ypmed.2014.08.028 25193005

[B38] Mattioli A. V. Sciomer S. Cocchi C. Maffei S. Gallina S. (2020). Quarantine during COVID-19 outbreak: Changes in diet and physical activity increase the risk of cardiovascular disease. Nutr. Metab. Cardiovasc. Dis. 30 (9), 1409–1417. 10.1016/j.numecd.2020.05.020 32571612 PMC7260516

[B39] McKenzie B. (2014). Modes less traveled—bicycling and walking to work in the United States: 2008–2012. [Online]. U.S. Census Bureau. Available at: https://usa.streetsblog.org/wp-content/uploads/sites/5/2014/05/acs-25 .pdf.

[B40] Morris J. N. Heady J. A. Raffle P. A. B. Roberts C. G. Parks J. W. (1953). Coronary heart-disease and physical activity of work. Lancet 262 (6795), 1053–1057. 10.1016/S0140-6736(53)90665-5 13110049

[B41] Owen N. Healy G. N. Matthews C. E. Dunstan D. W. (2010). Too much sitting: The population health science of sedentary behavior. Exerc. Sport Sci. Rev. 38 (3), 105–113. 10.1097/JES.0b013e3181e373a2 20577058 PMC3404815

[B42] O’Brien M. W. Wu Y. Petterson J. L. Frayne R. J. Kimmerly D. S. (2022). Ecological validity of prolonged sitting studies: How well do they represent real-life sedentary patterns? A pilot study. Transl. J. Am. Coll. Sports Med. 7 (1), e000182. 10.1249/tjx.0000000000000182

[B43] Owen N. Sugiyama T. Eakin E. E. Gardiner P. A. Tremblay M. S. Sallis J. F. (2011). Adults' sedentary behavior determinants and interventions. Am. J. Prev. Med. 41 (2), 189–196. 10.1016/j.amepre.2011.05.013 21767727

[B44] Padilla J. Sheldon R. D. Sitar D. M. Newcomer S. C. (2009). Impact of acute exposure to increased hydrostatic pressure and reduced shear rate on conduit artery endothelial function: A limb-specific response. Am. J. Physiol. Heart Circ. Physiol. 297 (3), H1103–H1108. 10.1152/ajpheart.00167.2009 19633210

[B45] Paterson C. Fryer S. Stone K. Zieff G. Turner L. Stoner L. (2021). The effects of acute exposure to prolonged sitting, with and without interruption, on peripheral blood pressure among adults: A systematic review and meta-analysis. Sports Med. 52, 1369–1383. 10.1007/s40279-021-01614-7 34932203

[B46] Paterson C. Fryer S. Zieff G. Stone K. Credeur D. P. Barone Gibbs B. (2020). The effects of acute exposure to prolonged sitting, with and without interruption, on vascular function among adults: A meta-analysis. Sports Med. 50 (11), 1929–1942. 10.1007/s40279-020-01325-5 32757163

[B47] Plummer T. Finestone E. (2017). “Archeological sites from 2.6 – 2.0 Ma: Towards a deeper understanding of the early Oldowan,” in Rethinking human evolution. Editor Schwartz J. (Cambridge: MIT Press).

[B48] Pontzer H. (2017). Economy and endurance in human evolution. Curr. Biol. 27 (12), R613–R621. 10.1016/j.cub.2017.05.031 28633035

[B49] Pontzer H. (2021). Hotter and sicker: External energy expenditure and the tangled evolutionary roots of anthropogenic climate change and chronic disease. Am. J. Hum. Biol. 33 (4), e23579. 10.1002/ajhb.23579 33629785

[B50] Pontzer H. Wood B. M. (2021). Effects of evolution, ecology, and economy on human diet: Insights from hunter-gatherers and other small-scale societies. Annu. Rev. Nutr. 41, 363–385. 10.1146/annurev-nutr-111120-105520 34138633

[B51] Pontzer H. Wood B. M. Raichlen D. A. (2018). Hunter-gatherers as models in public health. Obes. Rev. 19 (1), 24–35. 10.1111/obr.12785 30511505

[B52] Raichlen D. A. Pontzer H. Zderic T. W. Harris J. A. Mabulla A. Z. P. Hamilton M. T. (2020). Sitting, squatting, and the evolutionary biology of human inactivity. Proc. Natl. Acad. Sci. U. S. A. 117 (13), 7115–7121. 10.1073/pnas.1911868117 32152112 PMC7132251

[B53] Sarnat J. A. Golan R. Greenwald R. Raysoni A. U. Kewada P. Winquist A. (2014). Exposure to traffic pollution, acute inflammation and autonomic response in a panel of car commuters. Environ. Res. 133, 66–76. 10.1016/j.envres.2014.05.004 24906070 PMC4807398

[B54] Shaffer F. Ginsberg J. P. (2017). An overview of heart rate variability metrics and norms. Front. Public Health 5 (258). 10.3389/fpubh.2017.00258 PMC5624990 29034226

[B55] Stoner L. Barone Gibbs B. Meyer M. L. Fryer S. Credeur D. Paterson C. (2021). A primer on repeated sitting exposure and the cardiovascular system: Considerations for study design, analysis, interpretation, and translation. Front. Cardiovasc. Med. 8, 716938. 10.3389/fcvm.2021.716938 34485414 PMC8415972

[B56] Thomson M. Spence J. C. Raine K. Laing L. (2008). The association of television viewing with snacking behavior and body weight of young adults. Am. J. Health Promot. 22 (5), 329–335. 10.4278/ajhp.22.5.329 18517093

[B57] US Preventive Services Task Force (2014). Healthful diet and physical activity for cardiovascular disease prevention in adults with cardiovascular risk factors: Behavioral counseling. [Online]. Available at: https://www.uspreventiveservicestaskforce.org/uspstf/recommendation/healthy-diet-and-physical-activity-counseling-adults-with-high-risk-of-cvd (Accessed May 26, 2022).

[B58] U.S. Department of Health and Human Services (2022). *Move Your way* [online]. Office of disease prevention and health promotion. Available: https://health.gov/moveyourway .

[B59] U.S. Department of Health and Human Services (2018). Physical activity guidelines for Americans. [Online]. Washington, DC. Available: https://health.gov/sites/default/files/2019-09/Physical_Activity_Guidelines_2nd_edition.pdf (Accessed February 22, 2018).Office of disease prevention and health promotion

[B60] van der Ploeg H. P. Hillsdon M. (2017). Is sedentary behaviour just physical inactivity by another name? Int. J. Behav. Nutr. Phys. Act. 14 (1), 142. 10.1186/s12966-017-0601-0 29058587 PMC5651642

[B61] Walsh L. K. Restaino R. M. Martinez-Lemus L. A. Padilla J. (2017). Prolonged leg bending impairs endothelial function in the popliteal artery. Physiol. Rep. 5 (20), e13478. 10.14814/phy2.13478 29061865 PMC5661238

[B62] Ward B. W. Clarke T. C. Freeman G. Schiller J. S. (2015). Early release of selected estimates based on data from the 2014. *National Health Interview Survey.* [Online]. National Center for Health Statistics. Available at: https://www.cdc.gov/nchs/data/nhis/earlyrelease/earlyrelease201506_07.pdf .

[B63] Wood B. M. Harris J. A. Raichlen D. A. Pontzer H. Sayre K. Sancilio A. (2021). Gendered movement ecology and landscape use in Hadza hunter-gatherers. Nat. Hum. Behav. 5 (4), 436–446. 10.1038/s41562-020-01002-7 33398143 PMC8060163

